# Supplementary material for: The impact of maternal mood and economic stress during Covid-19 pandemic on infant behaviour: Findings from the cross-sectional UK Covid-19 New Mum Study
Source: PLOS Glob Public Health. 2024 Apr 17;4(4):e0003095. doi: 10.1371/journal.pgph.0003095 (PMC11023226; doi:10.1371/journal.pgph.0003095)
Supplement: S1 Table — Kaiser-Meyer-Olkin Measure of Sampling Adequacy = 0.885. Bartlett test of sphericity: p = <0.001. Rotation method: Varimax with Kaiser normalization. Component 1: Higher score reflect poor mental health. Component 2: Higher score reflect better coping. (DOCX) [file pgph.0003095.s001.docx]

S1 Table. Maternal wellbeing components from the principal component analysis (PCA); N= 2,031

|  | Components |
| --- | --- |
|  | **1. Maternal mental health** |
| Feeling down | 0.80 |
| Feeling lonely | 0.71 |
| Trouble relaxing | 0.81 |
| Easily annoyed | 0.71 |
| Feeling worried | 0.74 |
| Poor appetite | 0.41 |
| Trouble sleeping | 0.66 |
|  | **2. Coping and**  **focus on health and interests** |
| Opportunity to chat with family | 0.47 |
| Enjoy the weather | 0.58 |
| Enjoy personal interests | 0.60 |
| Focus on health | 0.77 |
| Time to exercise | 0.76 |

Kaiser-Meyer-Olkin Measure of Sampling Adequacy=0.899

Bartlett test of sphericity: p=<0.001

Rotation method: Varimax with Kaiser normalization

Component 1: Higher score reflect poor mental health.

Component 2: Higher score reflect better coping.
