# Supplementary material for: The impact of maternal mood and economic stress during Covid-19 pandemic on infant behaviour: Findings from the cross-sectional UK Covid-19 New Mum Study
Source: PLOS Glob Public Health. 2024 Apr 17;4(4):e0003095. doi: 10.1371/journal.pgph.0003095 (PMC11023226; doi:10.1371/journal.pgph.0003095)
Supplement: S2 Table — (DOCX) [file pgph.0003095.s002.docx]

S2 Table Univariable logistic regression analysis predicting infant behaviour

| Outcome | **Fussier** | | | | **Crying more** | | | |
| --- | --- | --- | --- | --- | --- | --- | --- | --- |
|  | OR | SE | p-value | 95%CI | OR | SE | p-value | 95%CI |
| ***Maternal mood and appetite and sleep changes*** | | | | | | | | |
| Feeling down | | | | | | | | |
| Not at all | *Reference group* | | | | | | | |
| Very little | 1.03 | 0.21 | 0.85 | 0.70;1.53 | 1.19 | 0.37 | 0.55 | 0.65;2.19 |
| To some extent | 1.51 | 0.28 | 0.03 | 1.04;2.18 | 1.57 | 0.46 | 0.12 | 0.89;2.79 |
| To a high extent | 2.88 | 0.56 | <0.001 | 1.96;4.22 | 3.30 | 0.96 | <0.001 | 1.86;5.85 |
| Feeling lonely | | | | | | | | |
| Not at all | *Reference group* | | | | | | | |
| Very little | 1.65 | 0.33 | 0.01 | 1.12;2.44 | 1.26 | 0.39 | 0.46 | 0.68;2.32 |
| To some extent | 2.02 | 0.37 | <0.001 | 1.42;2.89 | 1.95 | 0.54 | 0.02 | 1.12;3.37 |
| To a high extent | 3.84 | 0.69 | <0.001 | 2.69;5.48 | 3.98 | 1.07 | <0.001 | 2.35;6.77 |
| Easily annoyed | | | | | | | | |
| Not at all | *Reference group* | | | | | | | |
| Very little | 1.50 | 0.34 | 0.07 | 0.97;2.33 | 1.15 | 0.44 | 0.72 | 0.54;2.43 |
| To some extent | 2.27 | 0.47 | <0.001 | 1.51;3.42 | 2.58 | 0.88 | <0.01 | 1.31;5.05 |
| To a high extent | 3.93 | 0.82 | <0.001 | 2.60;5.92 | 5.28 | 1.79 | <0.001 | 2.71;10.28 |
| Trouble relaxing | | | | | | | | |
| Not at all | *Reference group* | | | | | | | |
| Very little | 1.69 | 0.36 | 0.01 | 1.12;2.55 | 1.00 | 0.33 | 0.98 | 0.53;1.89 |
| To some extent | 2.81 | 0.55 | <0.001 | 1.92;4.12 | 2.22 | 0.63 | <0.01 | 1.28;3.87 |
| To a high extent | 4.33 | 0.85 | <0.001 | 2.94;6.38 | 3.83 | 1.07 | <0.001 | 2.21;6.62 |
| Feeling worried | | | | | | | | |
| Not at all | *Reference group* | | | | | | | |
| Very little | 1.03 | 0.25 | 0.87 | 0.64;1.66 | 1.63 | 0.66 | 0.22 | 0.74;3.63 |
| To some extent | 1.17 | 0.26 | 0.46 | 0.76;1.81 | 1.77 | 0.68 | 0.14 | 0.83;3.76 |
| To a high extent | 1.95 | 0.43 | <0.01 | 1.27;3.01 | 3.13 | 1.19 | <0.01 | 1.49;6.60 |
| Poor appetite | | | | | | | | |
| Not at all | *Reference group* | | | | | | | |
| Very little | 1.56 | 0.20 | <0.01 | 1.21;2.00 | 1.79 | 0.32 | <0.01 | 1.26;2.53 |
| To some extent | 1.77 | 0.24 | <0.001 | 1.35;2.31 | 1.85 | 0.35 | <0.01 | 1.28;2.67 |
| To a high extent | 3.13 | 0.68 | <0.001 | 2.04;4.81 | 4.13 | 1.03 | <0.001 | 2.53;6.74 |
| Trouble sleeping | | | | | | | | |
| Not at all | *Reference group* | | | | | | | |
| Very little | 1.47 | 0.22 | <0.01 | 1.10;1.97 | 1.06 | 0.24 | 0.77 | 0.68;1.65 |
| To some extent | 2.27 | 0.32 | <0.001 | 1.73;2.99 | 1.86 | 0.37 | <0.01 | 1.26;2.74 |
| To a high extent | 2.82 | 0.42 | <0.001 | 2.11;3.78 | 2.89 | 0.58 | <0.001 | 1.95;4.28 |
| ***Impact on expenses*** | | | | | | | | |
| Impact on rent | | | | | | | | |
| No impact | *Reference group* | | | | | | | |
| Minor impact | 1.64 | 0.21 | <0.001 | 1.28;2.10 | 1.42 | 0.26 | 0.04 | 1.00;2.02 |
| Moderate/major impact | 1.47 | 0.19 | <0.01 | 1.14;1.89 | 1.97 | 0.33 | <0.001 | 1.43;2.73 |
| Food expenses | | | | | | | | |
| No impact | *Reference group* | | | | | | | |
| Minor impact | 1.61 | 0.20 | <0.001 | 1.26;2.05 | 2.27 | 0.38 | <0.001 | 1.63;3.15 |
| Moderate/major impact | 1.92 | 0.27 | <0.001 | 1.46;2.54 | 3.44 | 0.60 | <0.001 | 2.44;4.85 |
| Essentials | | | | | | | | |
| No impact | *Reference group* | | | | | | | |
| Minor impact | 1.44 | 0.17 | <0.01 | 1.11;1.85 | 1.79 | 0.31 | <0.01 | 1.27;2.53 |
| Moderate/major impact | 1.68 | 0.24 | <0.001 | 1.27;2.23 | 2.86 | 0.51 | <0.001 | 2.02;4.05 |
| ***Income*** | | | | | | | | |
| Over £100,000 | *Reference group* | | | | | | | |
| Less than £100,000 | 1.34 | 0.28 | 0.15 | 0.89;2.03 | 1.62 | 0.60 | 0.19 | 0.78;3.37 |
| Less than £75,000 | 1.84 | 0.33 | <0.01 | 1.29;2.61 | 2.51 | 0.81 | <0.01 | 1.33;4.72 |
| Less than £45,000 | 1.97 | 0.38 | <0.001 | 1.35;2.87 | 3.54 | 1.16 | <0.001 | 1.85;6.76 |
| Less than £30,000 | 2.91 | 0.62 | <0.001 | 1.91;4.41 | 5.81 | 1.98 | <0.001 | 2.97;11.35 |
| Less than £20,000 | 2.34 | 0.55 | <0.001 | 1.47;3.72 | 5.18 | 1.88 | <0.001 | 2.54;10.57 |

OR: Odds Ratios; SE: Standard error; CI: Confidence Interval
