# Supplementary material for: The impact of maternal mood and economic stress during Covid-19 pandemic on infant behaviour: Findings from the cross-sectional UK Covid-19 New Mum Study
Source: PLOS Glob Public Health. 2024 Apr 17;4(4):e0003095. doi: 10.1371/journal.pgph.0003095 (PMC11023226; doi:10.1371/journal.pgph.0003095)
Supplement: S3 Table — (DOCX) [file pgph.0003095.s003.docx]

S3 Table Univariable logistic regression analysis predicting infant behaviour

| Outcome | **Fussier** | | | | **Crying more** | | | |
| --- | --- | --- | --- | --- | --- | --- | --- | --- |
|  | OR | SE | p-value | 95%CI | OR | SE | p-value | 95%CI |
| ***Maternal coping*** | | | | | | | | |
| Feeling able to cope with the situation | | | | | | | | |
| Not at all | *Reference group* | | | | | | | |
| Very little | 0.27 | 0.06 | <0.001 | 0.18;0.41 | 0.30 | 0.08 | <0.001 | 0.17;0.51 |
| To some extent | 0.30 | 0.05 | <0.001 | 0.22;0.42 | 0.26 | 0.05 | <0.001 | 0.17;0.38 |
| To a high extent | 0.62 | 0.11 | <0.01 | 0.44;0.86 | 0.60 | 0.12 | 0.01 | 0.40;0.89 |
| Enjoying the weather | | | | | | | | |
| Not at all | *Reference group* | | | | | | | |
| Very little | 0.86 | 0.19 | 0.50 | 0.55;1.34 | 0.74 | 0.20 | 0.28 | 0.44;1.27 |
| To some extent | 0.48 | 0.10 | <0.01 | 0.32;0.73 | 0.38 | 0.09 | <0.001 | 0.23;0.63 |
| To a high extent | 0.40 | 0.09 | <0.001 | 0.27;0.61 | 0.32 | 0.08 | <0.001 | 0.19;0.53 |
| Opportunity to chat with family and friends | | | | | | | | |
| Not at all | *Reference group* | | | | | | | |
| Very little | 0.60 | 0.25 | 0.23 | 0.26;1.38 | 0.87 | 0.54 | 0.82 | 0.26;2.97 |
| To some extent | 0.82 | 0.34 | 0.64 | 0.36;1.88 | 1.03 | 0.64 | 0.96 | 0.30;3.50 |
| To a high extent | 1.20 | 0.53 | 0.68 | 0.50;2.84 | 1.76 | 1.13 | 0.37 | 0.50;6.19 |
| Enjoy hobbies | | | | | | | | |
| Not at all | *Reference group* | | | | | | | |
| Very little | 0.73 | 0.08 | <0.01 | 0.59;0.91 | 0.66 | 0.10 | 0.01 | 0.49;0.91 |
| To some extent | 0.54 | 0.09 | <0.001 | 0.38;0.76 | 0.55 | 0.14 | 0.02 | 0.34;0.91 |
| To a high extent | 0.82 | 0.23 | 0.51 | 0.47;1.44 | 0.71 | 0.29 | 0.42 | 0.32;1.60 |
| Focus on health | | | | | | | | |
| Not at all | *Reference group* | | | | | | | |
| Very little | 0.62 | 0.07 | <0.001 | 0.49;0.78 | 0.55 | 0.08 | <0.001 | 0.41;0.74 |
| To some extent | 0.42 | 0.06 | <0.001 | 0.32;0.56 | 0.32 | 0.07 | <0.001 | 0.21;0.49 |
| To a high extent | 0.48 | 0.11 | <0.001 | 0.31;0.76 | 0.51 | 0.16 | 0.03 | 0.27;0.95 |
| Time to exercise | | | | | | | | |
| Not at all | *Reference group* | | | | | | | |
| Very little | 0.78 | 0.09 | 0.05 | 0.61;0.99 | 0.63 | 0.11 | 0.01 | 0.58;1.08 |
| To some extent | 0.63 | 0.08 | <0.001 | 0.48;0.82 | 0.60 | 0.11 | 0.01 | 0.56;1.08 |
| To a high extent | 0.48 | 0.09 | <0.001 | 0.33;0.69 | 0.47 | 0.12 | 0.01 | 0.29;0.78 |
| ***House chores more equally divided*** | | | | | | | | |
| Not at all | *Reference group* | | | | | | | |
| Very little | 0.67 | 0.08 | <0.01 | 0.53;0.86 | 0.86 | 0.14 | 0.36 | 0.61;1.19 |
| To some extent | 0.68 | 0.09 | <0.01 | 0.53;0.88 | 0.61 | 0.12 | 0.01 | 0.42;0.89 |
| To a high extent | 0.45 | 0.08 | <0.001 | 0.31;0.64 | 0.59 | 0.15 | 0.03 | 0.36;0.96 |

OR: Odds Ratios; CI: Confidence Interval
