# Supplementary material for: The impact of maternal mood and economic stress during Covid-19 pandemic on infant behaviour: Findings from the cross-sectional UK Covid-19 New Mum Study
Source: PLOS Glob Public Health. 2024 Apr 17;4(4):e0003095. doi: 10.1371/journal.pgph.0003095 (PMC11023226; doi:10.1371/journal.pgph.0003095)
Supplement: Attachment — Submitted filename: Response to reviewers Oct 29.docx [file pgph.0003095.s004.docx]

29^th^ October 2023

Journal Requirements:

1. Please provide additional details regarding participant consent. In the ethics statement in the Methods and online submission information, please ensure that you have specified (1) whether consent was informed and (2) what type you obtained (for instance, written or verbal, and if verbal, how it was documented and witnessed). If your study included minors, state whether you obtained consent from parents or guardians. If the need for consent was waived by the ethics committee, please include this information.

We sought participant’s consent at the time that they selected the option to continue from the survey introductory webpage to the survey itself, as detailed below. Consistent with the requirements of our ethics committee, we have clarified that participants were being asked explicitly to consent to take part in the survey and to consent to allow UCL researchers to analyse the data to improve understanding of how the ‘lockdown and/or Covid-19 pandemic’ was affecting infant feeding practices.

We made clear that no personal identifier data were being collected, hence, participation in the survey was entirely anonymous. This also meant that participants would not be able to withdraw their data from the survey once they had completed it, as we would not be able to identify their entry. Despite the anonymity of the data, we also stated that we would use the data collected under the conditions set out by GDPR and UCL University data protection policies. All data would remain confidential, we would not share the raw data outside UCL.

We stated the following in the ‘Methods section’:

‘Ethical approval for the survey was obtained from the UCL Research Ethics committee (0326/017). The first page of the survey provided information about the study. Having read this, and the statements regarding the use of the anonymous data for research purposes, participants were asked to provide consent by ticking a box confirming their willingness to participate and for their data to be analysed by UCL researchers, before proceeding with subsequent pages of the survey. They were reminded not to provide any information in the free-text boxes that could allow identification. Despite the anonymity of the data, we also stated that we would use the data collected under the conditions set out by GDPR and UCL University data protection policies.’

If you are reporting a retrospective study of medical records or archived samples, please ensure that you have discussed whether all data were fully anonymized before you accessed them and/or whether the IRB or ethics committee waived the requirement for informed consent. If patients provided informed written consent to have data from their medical records used in research, please include this information.

We are not reporting a retrospective study on medical records of archive samples.

2. Please provide separate figure files in .tif or .eps format.

Figures were uploaded in .tif format at 300 dpi.

Reviewer #1: This study investigates the impact of maternal mood and economic stress during the early stages of the COVID19 pandemic on infant crying and fussiness in a sample of 2031 mother-infant dyads. Main results show that maternal wellbeing plays a significant role in children’s behavioural changes during the pandemic. Although the topic of the paper is interesting and the sample size is big, there are significant methodological issues that severely affect the quality of this study.

Thank you very much for your comments. Please find below the responses to your concerns.

Main concerns

- Data collection covers a wide time frame, from May 2020 to June (February?) 2021. Consequently, it is difficult to distinguish the effects of lockdown restrictions from those related to less stressful pandemic period on both maternal mental health and infant’s behaviour.

Thanks for pointing out the lack of clarity with the time frame. The Covid-19 New Mum Study was carried out from May 2020 to June 2021. To analyse changes in infant behaviour, we only used the data from those participants whose children were born before the Covid-19 pandemic. During all the study period, we obtained n= 3430 responses of which n=2,031 were from participants whose infants were born before the pandemic. Therefore, for our analysis we excluded n=1,399 responses.

The last data point that complies with our criterion, i.e., born before the pandemic, was captured in February 2021. However, most of the data used in our analysis was obtained between the end of May and August 2020 (n=1,997/2,031).

We added the following information in the ‘Methods’ section:

‘Covid-19 was confirmed to be spreading in the UK by the end of January 2020 and this was followed by three national lockdowns. The first was from 23^rd^ of March to 1^st^ of June (when stay-at-home orders were removed) followed by a period of 'relaxed' restrictions until October 2020. The second lockdown came into force on the 5^th^ of November 2020, and was due to end on the 26^th^ of November 2020. Followed by a partial easing of restrictions during December 2020, the UK entered a third lockdown between the 4^th^ of January and 29^th^ of March 2021 (https://www.instituteforgovernment.org.uk/sites/default/files/2022-12/timeline-coronavirus-lockdown-december-2021.pdf). The Covid-19 New Mum Study covered all these lockdown periods and the periods of easing of restrictions until June 2021. However, to analyse changes in infants’ behaviour attributable to the pandemic, we included only responses from mothers whose infants were born before the pandemic started. During the study period, a total of 3,430 women resident in the UK responded to the survey of which 2,031 were from participants whose infants were born before the pandemic. This data was captured between May 2020 and February 2021 (n= 2,031), with the vast majority of the data (98.3%) obtained between the 27th of May and the 28th of August 2020.’

We also provide clarification in the ‘Data’ section:

‘Data captured during the period 27^th^ May 2020 to 6^th^ February 2021 was used for the analysis, and the following variables were included in our analysis…’

- Mothers were asked to evaluate changes in behaviour of their infants as a result of staying home or the COVID-19 pandemic. Retrospective bias need to be considered, especially when participants were recruited one year after the outbreak of the COVID-19 pandemic.

Assuming that you are referring to recall bias, we added the following limitation:

‘Although maternal retrospective recall on infants' behaviour allowed us to explore behavioural changes after exposure to a unique and unpredictable phenomenon, the Covid-19 pandemic, it could also potentially introduce bias in the recollection process. However, most of the data used in our analysis was obtained between the end of May and August 2020 (n=1,997/2,031), at the beginning of the pandemic, hence exposure to the pandemic would have been relatively fresh in the participants memory.’

- No validated instruments were used to assess maternal psychosocial wellbeing and infant crying and fussing.

We did not specifically conduct a survey of mental ill-health or depression, rather from our survey we aimed to obtain a broader assessment of maternal mood and infant behaviour, incorporating both positive and negative aspects. This balanced approach was reflected in our interspersing of the positive questions and negative questions in our survey, and we omitted intrusive questions (e.g., relating to feelings of hopelessness, depression, self-harm, suicidal thoughts or violence in the home). We believe that by being willing to collect these data, we were indicating to the target population that we wanted to help, by learning how we might improve the support available. We offered a chance for mothers to tell us what was challenging them.

We have previously published these data on maternal mood (Dib S, Rougeaux E, Vázquez-Vázquez A, Wells JCK, Fewtrell M. Maternal mental health and coping during the COVID-19 lockdown in the UK: Data from the COVID-19 New Mum Study. Int J Gynecol \& Obstet. 2020;151(3):407–14), showing that a majority of > 1300 mothers reported feeling lonely, irritable and worried to some extent since lockdown began, but that the majority also felt able to cope. We believe that these data on maternal mood are very valuable for exploring how exposure to the pandemic influenced infant behaviour, through the mediation by maternal mood as analysed here.

We added the following information in the ‘Discussion’ section:

‘Moreover, we did not specifically survey maternal mental ill-health/depression and temperament in infants. Therefore, we did not formally assess maternal mental health and infant behaviour. Consequently, we were not able to compare the rates of depression or anxiety in new mothers or difficult behaviours in children, such as negative affectivity, during the COVID-19 pandemic with the rates before the pandemic. Through our survey, we wanted to learn the challenges that new mothers experienced as well as how to improve the support available.’

Reviewer #2: Thanks for giving me the opportunity to read this paper, which investigates an important topic concerning the link between maternal mental health during the lockdown and infant’s behavior in a large sample of 2031 families from the UK Covid-19 New Mum Study. Results revealed an association between maternal mental health, coping and financial strain and changes in infant’s behavior during the lockdown (i.e. crying more and being fussier). The manuscript is clear and nicely written. The topic is interesting and important. The large sample collected during a challenging time is a strength of the current paper. However, I have some concerns that temper enthusiasm for publication of the manuscript in its current form.

Thank you very much for your comments. Please find below the responses to your concerns.

Please find my major concerns better outlined below

1. Introduction: on page 3 it is stated that “the Covid-38 19 New Mum Study aimed to

capture information on maternal experiences and mood and infant feeding practices and

behaviour during the initial and subsequent stages of the lockdown”. Please specify whether prenatal or postnatal maternal experiences were the focus of the study and provide rationale for this. It might be important to refer also to available meta-analytical evidence showing that maternal mental health was significantly worsened from pre-pandemic period to during the pandemic (e.g., Tomfohr-Madsen et al., 2021)

We aimed to survey the experience of UK mothers feeding young infants during the Covid-19 pandemic, therefore, we collected postnatal information on maternal experiences and mood and infant feeding practices and behaviour during the initial and subsequent stages of the Covid-19 pandemic.

We obtained four types of information: (1) basic social and socio-economic household characteristics; (2) the impact of COVID-19 on household finances and work patterns; (3) infant feeding and behaviour and changes due to COVID-19; and (4) the impact of COVID-19 on the mother’s activities, mood and access to support. These data helped identify how COVID-19 was impacting infant feeding practices, which groups were most affected, and which were least able to access relevant support.

Our current analysis builds on some of the findings obtained in previous analysis that have been published and we wanted to test whether maternal mood and financial insecurity were associated with changes in infants' crying and fussiness during the pandemic.

We stated in our ‘Introduction’ and ‘Figure 1’ that ‘…using data from the Covid-19 New Mum Study and considering the findings in our previous analysis (1–3), we explored changes reported by the mother in specific behaviours of the infant (crying and fussiness) during the pandemic and to identify associations with maternal mood and financial insecurity (Fig 1).’

References:

1. Vazquez-Vazquez A, Dib S, Rougeaux E, Wells JC, Fewtrell MS. The impact of the Covid-19 lockdown on the experiences and feeding practices of new mothers in the UK: Preliminary data from the COVID-19 New Mum Study. Appetite [Internet]. 2021;156:104985. Available from: https://www.sciencedirect.com/science/article/pii/S019566632031607X
2. Dib S, Rougeaux E, Vázquez-Vázquez A, Wells JCK, Fewtrell M. Maternal mental health and coping during the COVID-19 lockdown in the UK: Data from the COVID-19 New Mum Study. Int J Gynecol \& Obstet [Internet]. 2020;151(3):407–14. Available from: https://obgyn.onlinelibrary.wiley.com/doi/abs/10.1002/ijgo.13397
3. Rougeaux E, Dib S, Vázquez-Vázquez A, Fewtrell MS, Wells JCK. Socio-economic impacts of the COVID-19 pandemic on new mothers and associations with psychosocial wellbeing: Findings from the UK COVID-19 New Mum online observational study (May 2020-June 2021). PLOS Glob Public Heal [Internet]. 2022;2(7):1–17. Available from: https://doi.org/10.1371/journal.pgph.0000576

In terms of the rationale of our analysis, we stated in the ‘Introduction’: ‘…Most previous research has focused on young children. However, infants merit particular attention as their experiences during this life stage may have effects on the bonding with their mothers, and the quality of this bonding can have long-term implications on their behaviour (17,18).’

Moreover, we stated in the ‘Statistical analysis’ section: ‘…For this analysis, we focused on negative changes in behaviours (more fussy and more crying then before the pandemic) to identify high-risk groups, i.e., groups that could need more help/support during challenging times such as the pandemic.’

We added more evidence as suggested. Please see the next comment.

2. On page 3 the authors reported “The impacts on maternal mental health may be of particular importance for early child development. Studies have suggested that maternal prenatal and postnatal stress could influence cognitive development and temperament in children (4–8) Since the Covid-19 outbreak, women have been exposed to a stressful situation of unknown duration that can be influencing their children’s behaviours (9–15).”. While it is important to mention this kind of broader literature, I think it is also important to acknowledge that a number of studied has already showed a link between maternal distress related to the pandemic and infant’s temperament and development, for example Provenzi et al., 2021; Nazzari et al., 2023, Giesbrecht et al., 2022).

Thank you for the suggestion. We rewrote a paragraph in the introduction and cited literature as follows:

‘Most previous research has focused on young children. However, infants merit particular attention as their experiences during this life stage may have effects on the bonding with their mothers, and the quality of this bonding can have long-term implications on their behaviour (16,17). For instance, Bianco et al (18) reported that prenatal maternal SARS-CoV-2 infection was not associated with infant temperament, but women who reported greater COVID-related life disruptions rated their infants higher on the negative emotionality IBQ-R subscale at 6 months of age. Moreover, higher maternal ratings of stress, as measured by the PSS at 4 months postpartum, were associated with lower values for infants’ positive affectivity/surgency and orienting/regulation capacity. Similarly, Provenzi et al reported that maternal prenatal stress due to the Covid-19 pandemic was associated indirectly with lower positive affectivity (19) and directly with lower regulatory capacity (20) in infants. However, it should be noted that at least one study reported no distinct impact of the pandemic on the typical development of temperament across infancy and early childhood (21).’

Literature added:

18. Bianco C, Sania A, Kyle MH, Beebe B, Barbosa J, Bence M, et al. Pandemic beyond the virus: maternal COVID-related postnatal stress is associated with infant temperament. Pediatr Res [Internet]. 2023 Jan 20;93(1):253–9. Available from: https://www.nature.com/articles/s41390-022-02071-2

19. Provenzi L, Mambretti F, Villa M, Grumi S, Citterio A, Bertazzoli E, et al. Hidden pandemic: COVID-19-related stress, SLC6A4 methylation, and infants’ temperament at 3 months. Sci Rep [Internet]. 2021 Aug 2;11(1):15658. Available from: https://www.nature.com/articles/s41598-021-95053-z

20. Provenzi L, Grumi S, Altieri L, Bensi G, Bertazzoli E, Biasucci G, et al. Prenatal maternal stress during the COVID-19 pandemic and infant regulatory capacity at 3 months: A longitudinal study. Dev Psychopathol [Internet]. 2023 Feb 2;35(1):35–43. Available from: https://www.cambridge.org/core/product/identifier/S0954579421000766/type/journal_article

Moreover, we added the following paragraph in the Discussion section:

‘In fact, our results are in line with a recent study published that women who reported higher perceived stress during the pandemic rated their infants lower in the positive affectivity/surgency and orienting/regulation scales, and those who reported a higher negative impact of the pandemic in their life rated their infants higher in the negative emotionality scale (18).’

3. I think it would be important to include in the introduction or method section a paragraph providing more context about the lockdown period in the UK in order to be able to better understand how it might have impact maternal and infant behaviour as well as draw comparisons with data from other countries.

We added in the methods section a brief timeline of UK coronavirus and measures during this period:

‘Covid-19 was confirmed to be spreading in the UK by the end of January 2020 and this was followed by three national lockdowns. The first was from 23rd of March to 1st of June (when stay-at-home orders were removed) followed by a period of 'relaxed' restrictions until October 2020. The second lockdown came into force on the 5th of November 2020, and was due to end on the 26th of November 2020. Followed by a partial easing of restrictions during December 2020, the UK entered a third lockdown between the 4th of January and 29th of March 2021 (https://www.instituteforgovernment.org.uk/sites/default/files/2022-12/timeline-coronavirus-lockdown-december-2021.pdf). The Covid-19 New Mum Study covered all these lockdown periods and the periods of easing of restrictions until June 2021. However, to analyse changes in infants’ behaviour attributable to the pandemic, we included only responses from mothers whose infants were born before the pandemic started. During the study period, a total of 3,430 women resident in the UK responded to the survey of which 2,031 were from participants whose infants were born before the pandemic. This data was captured between May 2020 and February 2021 (n= 2,031), with the vast majority of the data (98.3%) obtained between the 27th of May and the 28th of August 2020.’

4. Methods: the questionnaires employed in the online survey are not clearly mentioned, so I am not sure whether any kind of standardized instrument has been employed. As several widely employed and well-validated questionnaires exists to assess maternal mood, coping and infant behavior, I would like to know whether the authors employed any of this or reasons for not doing so.

As discussed in the response to Reviewer 1 above, we did not specifically conduct a survey of mental ill-health/depression and infant temperament, rather from our survey we aimed to obtain a broader assessment of maternal mood and infant behaviour, incorporating both positive and negative aspects. This balanced approach was reflected in our interspersing of the positive questions and negative questions in our survey, and we omitted intrusive questions (e.g., relating to feelings of hopelessness, depression, self-harm, suicidal thoughts or violence in the home). We believe that by being willing to collect these data, we were indicating to the target population that we wanted to help, by learning how we might improve the support available. We offered a chance for mothers to tell us what was challenging them.

We added, in the Methods section, the following information about our questionnaire:

‘The full content of the survey that includes details about the background factors, infant feeding practices, impacts of Covid-19 has been described elsewhere (1). Overall, we obtained four types of information: (1) basic social and socio-economic household characteristics; (2) the impact of COVID-19 on household finances and work patterns; (3) infant feeding and behaviour and changes due to COVID-19; and (4) the impact of COVID-19 on the mother’s activities, mood and access to support. These data helped identify how COVID-19 was impacting infant feeding practices, which groups were most affected, and which were least able to access relevant support. For the present study, to explore changes in infant behaviour due to the lockdown and/or the pandemic, we only include the responses provided by mothers whose children were born before 23rd March 2020 (n=2,031).’

Reference 1: Vazquez-Vazquez A, Dib S, Rougeaux E, Wells JC, Fewtrell MS. The impact of the Covid-19 lockdown on the experiences and feeding practices of new mothers in the UK: Preliminary data from the COVID-19 New Mum Study. Appetite [Internet]. 2021;156:104985. Available from: <https://www.sciencedirect.com/science/article/pii/S019566632031607X>.

Furthermore, we added the following information in the ‘Discussion’ section:

‘Moreover, we did not specifically survey maternal mental ill-health/depression and temperament in infants. Therefore, we did not formally assess maternal mental health and infant behaviour. Consequently, we were not able to compare the rates of depression or anxiety in new mothers or difficult behaviours in children, such as negative affectivity, during the COVID-19 pandemic with the rates before the pandemic. Through our survey, we wanted to learn the challenges that new mothers experienced as well as how to improve the support available.’

5. Results: the authors computed two principal components respectively for maternal mental health and coping, however in the analyses reported on page 10 and 11 they run univariable logistic regression including all items (I guess) of the survey as possible predictors of infant behaviour. Thus the analytical strategy is not really entirely clear to me.

First, we identified if any of the variables could explain a significant fraction of the variance in the data. Once we had established if any of the variables can explain some of the variability, then we could to assess how they interacted together, i.e., if they were colinear, or correlated with each other, etc. Simple associations of each predictor with the outcomes were statistically significant (except for ‘Opportunity to chat with family and friends’ in Table S3).

We added the following information in the methods section:

‘We used univariate logistic analysis to test the simple association of each predictor with the outcome. We then conducted multivariate logistic analysis to test the associations when controlling for all other predictors.’

We also stated in the ‘Results’ section:

‘Univariable logistic analyses of maternal or household economic predictors of infant behaviour are shown in S2 Table and S3 Table. Overall, women who experienced negative changes in their mood and difficulties in their ability to cover household expenses had higher odds of reporting difficult infant behaviour during lockdown (S2 Table). Conversely, women who reported being able to cope with the situation and to enjoy personal interests and focus on their own health had lower odds of reporting difficult infant behaviour (S3 Table). An example of this analysis is shown in Fig 2. Women who reported having trouble relaxing, feeling lonely or experiencing difficulties meeting food expenses had significantly higher odds of reporting that infants were fussier (Figs 2a, b and c). In contrast, women who were able to focus on their own health had significantly lower odds of reporting that infants were fussier (Fig 2d).’

6. Please follow APA style to report statistical results both in text and tables (e.g., β, SE, p-values are missing)

Done

7. Please provide model significance of multivariable logistic regression analysis.

Done

8. Considering the large sample why did the authors did not choose to test any moderation/mediation model? For example, it might be interesting to test whether financial strain might further exacerbate the impact of maternal mental health on infant behavior as well as whether maternal coping might buffer the impact of adverse conditions (e.g. high financial stress or low maternal mood).

We did not carry out a mediation analysis because examining mediation in cross-sectional data implicitly undermines an assumption of the statistical mediation model: the presumption that temporal ordering of variables in the causal chain of mediation is correct. All our data were collected at the same time point, making this kind of analysis inappropriate.

9. The authors stated that “Moreover, we did not formally assess maternal mental health and infant behaviour due to the anonymous nature of the survey which made it not possible to identify participants for follow up” – this sentence is not clear and need to be rephrased

We rephrased and added the following information in the ‘Discussion’ section:

‘Moreover, we did not specifically survey maternal mental ill-health/depression and temperament in infants. Therefore, we did not formally assess maternal mental health and infant behaviour. Consequently, we were not able to compare the rates of depression or anxiety in new mothers or difficult behaviours in children, such as negative affectivity, during the COVID-19 pandemic with the rates before the pandemic. Through our survey, we wanted to learn the challenges that new mothers experienced as well as how to improve the support available.’

10. Figures are of low quality and need to be graphically improved

Figures were uploaded in .tif format at 300 dpi.
